# Glances at the Hospitals

**Published:** 1900-01-27

**Authors:** 


### GLANCES AT THE HOSPITALS.

### THE MONKWEARMOUTH AND SOUTHWICK HOSPITAL.

DURING the year 1899 the number of patients treated in this hospital was 5,941, being a considerable decrease on the number treated in 1898. Table I. shows that this decrease was among the out-patients only; in fact there were 567 in-patients, as against 452 during the previous year. As regards the decrease in the number of out-patients, the medical staff say they view this decrease with pleasure, and they add that in some large hospitals the out-patients' department has been entirely given up, and that "when there are so many agencies by which the poorer classes can command medical advice and treatment for ordinary routine cases at a very low rate of payment, it is not desirable that a hospital should, to any considerable extent, be hampered by dispensary work." This is a very rational way of looking at the question, and we hope the next annual report will show a still further diminution of these patients, not only as regards dispensary cases, but of those also who can afford to pay for private advice. The history of the twelve months shows that 347 cases of accidents were treated, in addition to 154 other surgical cases, and only 66 medical patients were admitted. The average duration of hospital treatment was 20 days. The death-rate was 3·17 per cent., and this included six cases admitted in a dying condition. An analysis of the accidents shows that there were 220 fractures, and of these 12 were cases of fracture of the skull and 1 of the pelvis. Nine amputations were performed, and all were successful. There was 1 unsuccessful case of nephrectomy and 1 successful case of nephrotomy. Atogether the major operations reached the respectable figure of 188, and the minor 260. The actual cost of carrying on the hospital during 1899 was £2,046, and the total income was only £1,947, showing a deficit of about £99. The work-people's own subscriptions amounted to £1,136—a very praiseworthy condition of affairs. Sir Hedworth Williamson, Bart., is president of the hospital, and the honorary medical and surgical staff consists of Drs. Coke, Stobo, Pearcey, Cox, Modlin, and Davis. For the resident medical staff the hospital seems a nursery for other institutions. Three of the house surgeons have obtained posts in the Liverpool and Sunderland Hospitals, and we may soon hope to learn that the present incumbent, Dr. Wilkie Scott, has been promoted to a wider sphere of usefulness.

